# Genetic Characteristics of Multidrug-Resistant *Salmonella* Isolated from Poultry Meat in South Korea

**DOI:** 10.3390/microorganisms12081646

**Published:** 2024-08-11

**Authors:** Haiseong Kang, Hansol Kim, Jonghoon Lee, Ji Hye Jeon, Seokhwan Kim, Yongchjun Park, Insun Joo, Hyochin Kim

**Affiliations:** 1 Food Microbiology Division, Food Safety Evaluation Department, National Institute of Food and Drug Safety Evaluation, Cheongju 28159, Republic of Korea; rkdgotjd12@korea.kr (H.K.); hskmfds@korea.kr (H.K.); whdgns46@korea.kr (J.L.); jj9099@korea.kr (J.H.J.); yongchjun@korea.kr (Y.P.); jis901@korea.kr (I.J.); 2 Food Standard Division, Ministry of Food and Drug Safety, Cheongju 28159, Republic of Korea; myksh@korea.kr

**Keywords:** antimicrobial resistance, multidrug-resistance, poultry meat, *Salmonella* spp., whole-genome sequencing

## Abstract

Given the lack of genetic characterization data for multidrug-resistant (MDR) *Salmonella* in South Korean poultry, we analyzed 53 MDR *Salmonella* strains from 1232 poultry meat samples (723 chicken, 509 duck) using whole-genome sequencing. Five serotypes were identified: *S.* Infantis (30/53, 56.6%), *S.* Enteritidis (11/53, 20.8%), *S.* Virchow (9/53, 17.0%), *S.* Agona (2/53, 3.8%), and *S.* Indiana (1/53, 1.9%). Sequence types (STs) included ST32, ST11, ST16, ST13, and ST17, with three major clusters, each having two subclusters. Eight core genome sequence types (cgSTs) were identified: 225993, 2268, 58360, 150996, 232041, 96964, 117577, and 267045. *Salmonella* Infantis and *S.* Enteritidis had two (117577, 267045) and three (225993, 2268, 58360) cgSTs, respectively, whereas *S.* Virchow showed allelic differences in identical cgSTs. The *S.* Enteritidis subcluster was classified as chicken or duck. Twenty-eight antimicrobial resistance genes (ARGs), 10 plasmid replicons, 11 *Salmonella* pathogenicity islands (SPIs), and 230 virulence genes were identified, showing distinct profiles by cluster and subcluster. *Salmonella* Infantis, the primary MDR *Salmonella*, carried the IncFIB (pN55391) plasmid, 10–11 ARGs, nine SPIs, and approximately 163 virulence genes. Three major MDR *Salmonella* serotypes (*S.* Infantis, *S.* Enteritidis, and *S.* Virchow) had specific genetic profiles that can inform epidemiological surveillance.

## 1. Introduction

*Salmonella* is the major etiological agent of diarrheal diseases, presenting an important worldwide health concern with 1.9 billion cases annually [1]. Poultry and egg products are the primary sources of *Salmonella* infection [2]. This phenomenon is also observed in South Korea. According to the Korean Ministry of Food and Drug Safety (MFDS), *Salmonella* is the third most common foodborne pathogen associated with foodborne illnesses in South Korea over the past two decades [3].

Various antimicrobials are widely used for disease prevention and treatment in the global livestock industry [4]. However, continuous exposure to antimicrobials decreases microbial diversity and increases the number of antimicrobial-resistant bacteria via selective pressure [5]. Therefore, livestock products continuously exposed to antimicrobials are exposed to the threat of antimicrobial-resistant bacteria. Livestock contaminated with antimicrobial-resistant bacteria serve as reservoirs and ensure transmission to the community through food [6]. Several studies have shown a strong causal link between antimicrobial usage in livestock and the emergence of antimicrobial resistance (AR) in pathogenic bacteria that cause human diseases [7,8].

Whole-genome sequencing (WGS) has become an affordable, high-resolution method for genome analyses, providing crucial information such as antimicrobial resistance genes (ARGs), genomic mutations, multilocus sequence typing (MLST), and core genome MLST (cgMLST) [9,10,11]. Thus, WGS is a useful method for tracking the source of foodborne diseases and confirming the transmission of *Salmonella* infections between poultry sources and humans [10,11].

Although multidrug-resistant *Salmonella* has been reported in poultry, genetic characterization data remain limited. Therefore, in this study, we aimed to isolate multidrug-resistant *Salmonella* spp. from South Korean poultry meat and analyze their genetic characteristics using WGS. In this study, we isolated *Salmonella* from poultry meat and monitored its susceptibility to 16 agents of 13 antimicrobial subclasses, including beta-lactams. In addition, phylogenetic analyses, including cgMLST of multidrug-resistant *Salmonella* and analysis of AR, plasmid, *Salmonella* pathogenicity island (SPI), mobile genetic element (MGE), and virulent factor (VF) genes, were performed. Our findings provide valuable data to enhance the understanding of foodborne, multidrug-resistant *Salmonella* in South Korean poultry meat, focusing on AR and virulence mechanisms.

## 2. Materials and Methods

### 2.1. Sample Collection

Between February 2020 and November 2021, poultry samples (723 chicken and 509 duck meat) were collected from retail markets in Korea (Table 1). Samples were collected from five regions. The samples were purchased from various companies, weighed between 200 g and 3 kg, and were immediately refrigerated and transported.

### 2.2. Salmonella Isolation and Identification

*Salmonella* spp. were isolated using an analytical method certified by the MFDS Food Code [12]. Briefly, approximately 25 g of the sample was dispensed with 225 mL of buffered peptone water (BPW, Merck, Darmstadt, Germany) into a sterilized blender bag, homogenized for 30 s, and incubated at 37 °C for approximately 24 h. Then, 0.1 mL and 1 mL of incubated BPW were transferred into 10 mL of Rappaport–Vassiliadis broth (BD, Franklin Lakes, NJ, USA) and 10 mL of tetrathionate broth (MBcell, Seoul, Republic of Korea), and incubated at 42 °C and 37 °C for 24 h, respectively. After incubation, each culture solution was spread on xylose lysine deoxycholate agar (XLD; Oxoid, Basingstoke, UK) as well as Brilliant Green Sulfa Agar (Remel, Lenexa, UK) and incubated at 37 °C for 24 h. The presumed *Salmonella* colonies were selected, spread on Tryptone Soya Agar (Oxoid), and incubated at 37 °C for 24 h. After incubation, the bacteria were identified at the species level using matrix-assisted laser desorption ionization-time of flight. At least one *Salmonella* isolate per sample was selected for further analysis. *Salmonella* isolates were stored at −80 °C in Tryptic Soy Broth (Oxoid) with 10% glycerol.

### 2.3. Antimicrobial Susceptibility

All identified *Salmonella* strains were subjected to an antimicrobial minimal inhibitory concentration (MIC) assay. The MIC test was performed using the KRNV5F (TREK Diagnostic Systems, Cleveland, OH, USA) panel for this assay according to the manufacturer’s instructions. *E. coli* ATCC 25922 was used as the reference strain. Each panel contained a total of 16 agents of 13 antimicrobial subclasses (Table 2). The MIC results were interpreted according to the breakpoint guidelines of the Clinical and Laboratory Standards Institute [13]. As the CLSI has no breakpoint guidelines for ceftiofur and streptomycin, these data were interpreted according to the National Antimicrobial Resistance Monitoring System [14].

### 2.4. WGS Analysis

Based on the MIC assay, strains resistant to five or more antimicrobial classes (n = 53) were selected for the WGS analysis. The selected strains were subjected to WGS at Senigen, Inc. (Seoul, Republic of Korea). Briefly, a MagListo 5M Genomic DNA Extraction Kit (Bioneer, Daejeon, Republic of Korea) was used for DNA extraction according to the manufacturer’s instructions. WGS was analyzed using an Illumina MiSeq desktop sequencer (Illumina Inc., San Diego, CA, USA) with paired-end reads of approximately 300 bp in length. Trimmomatics (version 0.38) was used for the trimming process. SPAdes (version 3.13.0) was used to assemble raw reads. The assembled sequence data were filtered out with a length of 1000 bp and a depth of at least 5. The assembled contig number ranged between 21 and 66 and from 101 to 236 with average depths of 39 and 153, respectively.

### 2.5. Serotyping and Homology Analysis

*Salmonella* serotypes were determined using SeqSero (version 1.2) [15]. Additionally, bacteria whose serotypes were not confirmed using SeqSero were tested using a slide agglutination test according to the Kauffman–White scheme using commercially available antisera (S&A Reagents Lab, Bangkok, Thailand). Homologies were compared using MLST and cgMLST. Seven housekeeping genes (*aroC*, *dnaN*, *hemD*, *hisD*, *purE*, *sucA*, and *thrA*) were obtained from the MLST database [16]. MLST (version 2.0) was used in silico on the Center for Genomic Epidemiology (CGE) website to determine the sequence type. For cgMLST, we used the cgMLSTfinder (version 1.2) on the CGE website to predict allelic profiles. A cgMLST-based minimum-spanning tree was constructed using GrapeTree (version 1.5.0).

### 2.6. In Silico Characterization of WGS

The genetic characterization of *Salmonella* was performed using WGS. ARGs were identified using ResFinder (version 4.1), with minimum identity and coverage thresholds set at 90% and 60%, respectively. Plasmid types and *Salmonella* pathogenicity islands (SPIs) were predicted using Plasmidfinder (version 2.1) and SPIFinder (version 2.0), with minimum identity and coverage thresholds of 95% and 60%, respectively. Mobile genetic elements (MGEs) were identified using mobile element finder (software version 1.0.3 and database version 1.0.2) [17], with minimum identity and coverage thresholds of 90% each. Virulence factors were predicted using the Virulence Factor Database [18], with minimum identity and coverage thresholds set at 90% and 50%, respectively.

### 2.7. Nucleotide Sequence Accession Numbers

The raw WGS data were deposited in GenBank under BioProject PRJNA1105733, with the biosample accession number SAMN41108762 (2020_64), SAMN41108763 (2020_352), SAMN41108764 (2020_572), SAMN41108765 (2020_975), SAMN41108766 (2020_997), SAMN41108767 (2020_1205), SAMN41108768 (2020_1435), SAMN41108769 (2021_277), SAMN41108770 (2021_360), SAMN41108771 (2021_436), SAMN41108772 (2021_623), SAMN41108773 (2020_1362), SAMN41108774 (2020_1395), SAMN41108775 (2020_378), SAMN41108776 (2020_422), SAMN41108777 (2020_475), SAMN41108778 (2020_537), SAMN41108779 (2020_661), SAMN41108780 (2020_890), SAMN41108781 (2020_1459), SAMN41108782 (2020_1513), SAMN41108783 (2021_1241), SAMN41108784 (2021_1567), SAMN41108785 (2020_354), SAMN41108786 (2020_357), SAMN41108787 (2020_760), SAMN41108788 (2020_1241), SAMN41108789 (2020_1396), SAMN41108790 (2020_1399), SAMN41108791 (2020_1400), SAMN41108792 (2020_1401), SAMN41108793 (2020_1403), SAMN41108794 (2020_1458), SAMN41108795 (2020_1509), SAMN41108796 (2021_16), SAMN41108797 (2021_430), SAMN41108798 (2021_486), SAMN41108799 (2021_563), SAMN41108800 (2021_761), SAMN41108801 (2021_849), SAMN41108802 (2021_888), SAMN41108803 (2021_932), SAMN41108804 (2021_1100), SAMN41108805 (2020_1357), SAMN41108806 (2021_1362), SAMN41108807 (2021_1429), SAMN41108808 (2021_1479), SAMN41108809 (2021_1500), SAMN41108810 (2021_1584), SAMN41108811 (2021_1648), SAMN41108812 (2021_1726), SAMN41108813 (2021_1741), and SAMN41108814 (2021_1759).

## 3. Results

### 3.1. Prevalence of Salmonella and MDR Salmonella in Poultry Meat Samples

The prevalence rates of *Salmonella* were 27.4% (n = 198) and 41.3% (n = 210) in 723 and 509 chicken and duck meats, respectively; 46.0% (n = 91) and 28.1% (n = 59) of *Salmonella* isolated from chicken and duck meats were multidrug-resistant (MDR; resistant to three or more antimicrobial classes) [19]. Of the 113 *Salmonella* resistant to at least five antimicrobial classes, 53 strains were selected and subjected to WGS analysis.

### 3.2. Serotyping and Phylogenetic Analysis

**Figure 1:**
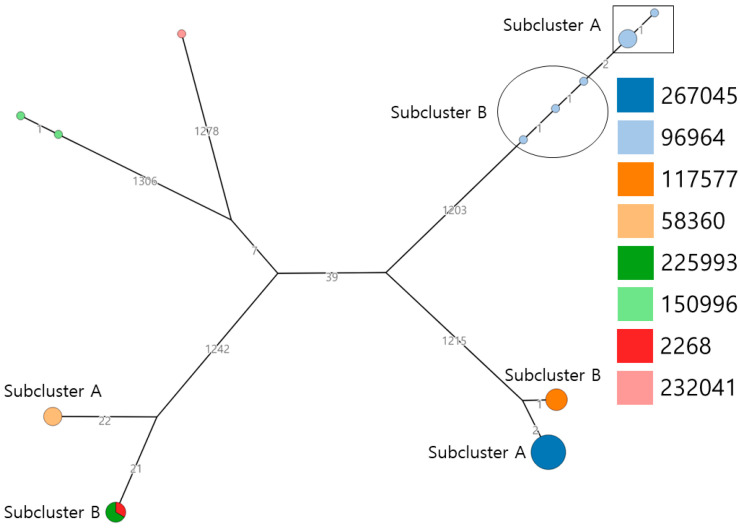
Core genome multilocus sequence typing based minimum-spanning tree.

Of the 53 tested *Salmonella*, five different *Salmonella* serovars were identified: *S.* Infantis (30/53, 56.6%), *S.* Enteritidis (11/53, 20.8%), *S.* Virchow (9/53, 17.0%), *S.* Agona (2/53, 3.8%), and *S.* Indiana (1/53, 1.9%). These five *Salmonella* serotypes belonged to distinct sequence types: *S*. Infantis, *S*. Enteritidis, *S*. Virchow, *S*. Agona, and *S*. Indiana belonged to ST32, ST11, ST16, ST13, and ST17, respectively. Fifty-three *Salmonella* isolates were identified from eight core genome sequence types (cgSTs) (Figure 1). *Salmonella* Infantis isolates were identified in two cgSTs (eight cgST117577 and 22 cgST267045), *S*. Enteritidis was identified in three cgSTs (four cgST225993, two cgST2268, and five cgST58360), *S*. Virchow was identified in cgST96964, *S*. Agona was identified in cgST150996, and *S*. Indiana was identified in cgST232041. Each serotype was divided into four clusters (*S*. Infantis, *S*. Enteritidis, *S*. Virchow, and *S*. Agona) and one singleton (*S*. Indiana). Three clusters (*S*. Infantis, *S*. Enteritidis, and *S*. Virchow) were further classified into two subclusters each. *Salmonella* Infantis was classified based on cgSTs (cgST117577 and cgST267045). *Salmonella* Enteritidis clusters were classified based on cgST and origin (i.e., subcluster A was identified as cgST58360 and was isolated from chicken meat, whereas subcluster B was identified as cgST225993 and cgST2268 and was isolated from duck meat). The *S*. Virchow cluster was separated based on allelic differences. 

### 3.3. Antimicrobial Resistance Patterns of MDR Salmonella 

**Figure 2:**
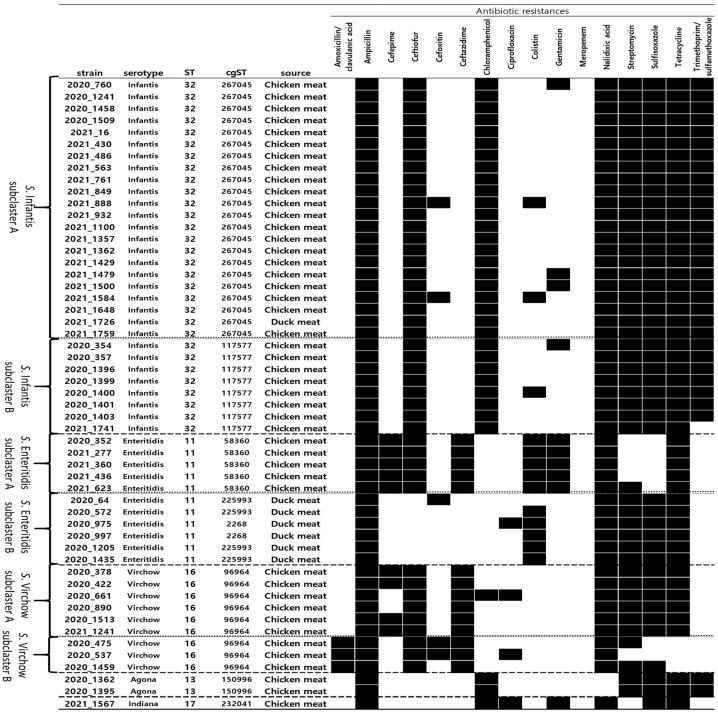
Antimicrobial resistance profiles of multidrug-resistant *Salmonella* spp.

The five serotypes showed different AR profiles, with *S*. Enteritidis and *S*. Virchow subclusters showing notable differences (Figure 2). Both A and B clusters of *S*. Infantis were resistant to seven antimicrobial classes (aminoglycoside, aminopenicillin, cephalosporin III, folate pathway inhibitor, phenicol, quinolone, and tetracycline). No specific differences in AR were observed between the *S*. Infantis subclusters. Both A and B clusters of *S*. Enteritidis were resistant to three antimicrobial classes (aminopenicillin, quinolone, and tetracycline). In addition, *S*. Enteritidis subclusters showed clear differences in resistance to four antimicrobial classes (aminoglycosides, cephalosporin III, cephalosporin IV, and folate pathway inhibitors). Both A and B clusters of *S*. Virchow were resistant to three antimicrobial classes (aminopenicillin, cephalosporin III, and quinolone). In addition, *S*. Virchow subclusters showed clear differences in resistance to two antimicrobial classes (β-lactam/β-lactamase inhibitor combination and tetracycline). Both A and B clusters of *S*. Virchow were resistant to three antimicrobial classes (aminopenicillin, cephalosporin III, and quinolone). In addition, *S*. Virchow subclusters showed clear differences in resistance to two antimicrobial classes (β-lactam/β-lactamase inhibitor combination and tetracycline). The *S*. Agona cluster was resistant to five antimicrobial classes (aminoglycoside, aminopenicillin, folate pathway inhibitor, phenicol, and tetracycline). The *S*. Indiana singleton was resistant to seven antimicrobial classes (aminoglycoside, aminopenicillin, fluoroquinolone, folate pathway inhibitor, phenicol, quinolone, and tetracycline).

### 3.4. Detection of Antimicrobial Resistance Genes, Plasmid Genes, Salmonella Pathogenicity Island, and Mobile Genetic Elements

**Figure 3:**
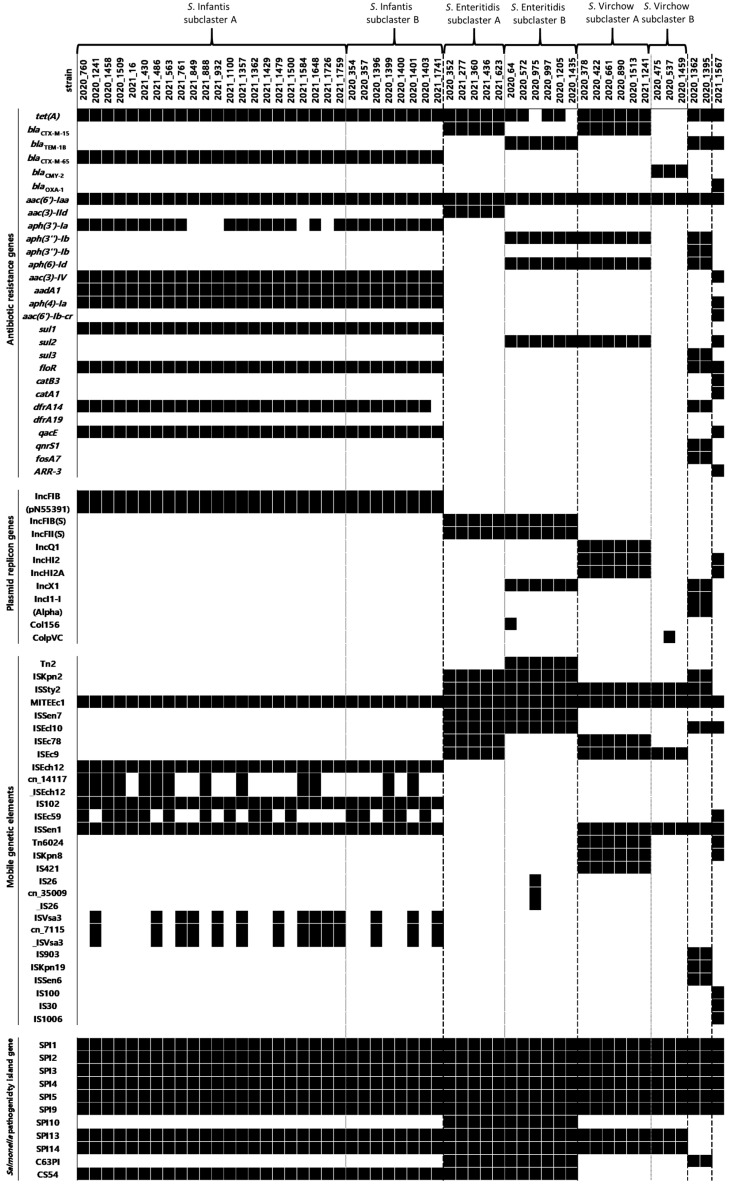
Identified antimicrobial resistance, plasmid, *Salmonella* pathogenicity island, and mobile genetic element gene profiles of multidrug-resistant *Salmonella* spp.

In this study, 10 classes of ARGs (beta-lactam, tetracycline, aminoglycoside, sulfonamide, phenicol, trimethoprim, disinfectant, quinolone, fosfomycin, and rifampicin) were revealed (Figure 3). In this study, *aac(6′)-Iaa* (53/53) was the most commonly detected gene. The *S*. Infantis subcluster A carried *tet(A)*, *bla*_CTX-M-65_, *aac(6′)-Iaa*, *aph(3′)-Ia* (17/22), *aac(3)-IV*, *aadA1*, *aph(4)-Ia*, *sul1*, *floR*, *dfrA14*, and *qacE*; *S*. Infantis subcluster B carried *tet(A)*, *bla*_CTX-M-65_, *aac(6′)-Iaa*, *aph(3′)-Ia*, *aac(3)-IV*, *aadA1*, *aph(4)-Ia*, *sul1*, *floR*, *dfrA14* (7/8), and *qacE; S*. Enteritidis subcluster A carried *tet(A)*, *bla*_CTX-M-15_, *aac(6′)-Iaa*, and *aac(3)-IId*; *S*. Enteritidis subcluster B carried *tet(A)* (4/6), *bla*_TEM-1B_, *aac(6′)-Iaa*, *aph(3*″*)-Ib*, *aph(6)-Id*, and *sul2*; *S*. Virchow subcluster A carried *tet(A)*, *bla*_CTX-M-15_, *aac(6′)-Iaa*, *aph(3*″*)-Ib*, *aph(6)-Id*, and *sul2; S*. Virchow subcluster B carried *bla*_CMY-2_, and *aac(6′)-Iaa; S*. Agona carried *tet(A)*, *bla*_TEM-1B_, *aac(6′)-Iaa*, *aph(3*″*)-Ib*, *aph(3*″*)-Ib*, *aph(6)-Id*, *sul3*, *floR*, *dfrA14*, *qnrS1*, and *fosA7;* and *S*. Indiana carried *tet(A)*, *bla*_TEM-1B_, *bla*_OXA-1_, *aac(6′)-Iaa*, *aac(3)-IV*, *aph(4)-Ia*, *aac(6′)-Ib-cr*, *sul2*, *floR*, *catB3*, *catA1*, *qacE*, and *ARR-3*. 

Moreover, 10 plasmid replicon types were identified, i.e., IncFIB (pN55391) (reference accession no: CP016411), IncFIB(S) (FN432031), IncFII(S) (CP000858), IncQ1 (M28829), IncHI2 (BX664015), IncHI2A (BX664015), IncX1 (JN935898, EU370913), IncI1-I(Alpha) (AP005147), Col156 (NC009781), and ColpVC (JX133088) (Figure 3). All *S.* Infantis strains were carried with IncFIB (pN55391); all *S*. Enteritidis isolates harbored both IncFIB(S) and IncFII(S); *S*. Enteritidis subcluster B also carried IncX1; *S*. Virchow subcluster A carried IncQ1, IncHI2, and IncHI2A; *S*. Virchow subcluster B did not contain any of the three plasmids; all *S*. Agona isolates harbored IncI1-I(Alpha) and IncX1; and *S*. Indiana carried both IncHI2 and IncHI2A. 

A total of 26 MGEs were identified: *S*. Infantis subcluster A was identified with MITEEc1, ISEch12, cn_14117_ISEch12 (11/22), IS102, ISEc59 (11/22), ISSen1, ISVsa3 (11/22), and cn_7115_ISVsa3 (11/22); *S*. Infantis subcluster B was identified with MITEEc1, ISEch12, cn_14117_ISEch12 (2/8), IS102, ISEc59 (5/8), ISSen1, ISVsa3 (3/8), and cn_7115_ISVsa3 (3/8); 14 *S*. Infantis were detected to carry *floR* within cn_7115_ISAsa3; *S*. Enteritidis subcluster A was identified with ISKpn2, ISSty2, MITEEc1, ISSen7, ISEcl10, ISEc78, and ISEc9; *S*. Enteritidis subcluster B was identified with Tn2, ISKpn2, ISSty2, MITEEc1, ISSen7, and ISEcl10; five *S*. Enteritidis subcluster B were detected to carry *bla*_TEM-1B_ with Tn2 and one strain was detected four AMR genes (*bla*_TEM-1B_, *aph(3*″*)-Ib*, *aph(6)-Id*, and *sul2*) and the IncX1 plasmid gene with cn_35009_IS26; *S*. Virchow subcluster A was identified with ISSty2, MITEEc1, ISEc78, ISEc9, ISSen1, Tn6024, ISKpn8, and IS421; *S*. Virchow subcluster B was identified with ISSty2, MITEEc1, ISEc9, and ISSen1; *S*. Agona was identified with ISKpn2, ISSty2, MITEEc1, ISEcl10, ISSen1, IS903, ISKpn19, and ISSen6; and *S*. Indiana was identified with MITEEc1, ISEcl10, ISEc59, ISSen1, Tn6024, ISKpn8, IS100, and IS30.

Each *Salmonella* serotype carried identical pathogenicity island genes (Figure 3). *Salmonella* Infantis, *S*. Enteritidis, *S*. Virchow, *S*. Agona, and *S*. Indiana carried nine, 11, eight, seven, and six SPI genes, respectively. Eleven SPI genes were identified, and six SPI genes (*SPI1*, *SPI2*, *SPI3*, *SPI4*, *SPI5*, and *SPI9*) were found to be common in this study. Two SPI genes (*SPI13* and *SPI14*) were commonly found in *S*. Infantis, *S*. Enteritidis, and *S*. Virchow. *Salmonella* Infantis, *S*. Enteritidis, and *S*. Agona carried one (*CS54*), three (*SPI10*, *C63PI*, and *CS54*), and one (*C63PI*) gene, respectively.

### 3.5. Detection of Virulence Factor Genes

**Figure 4:**
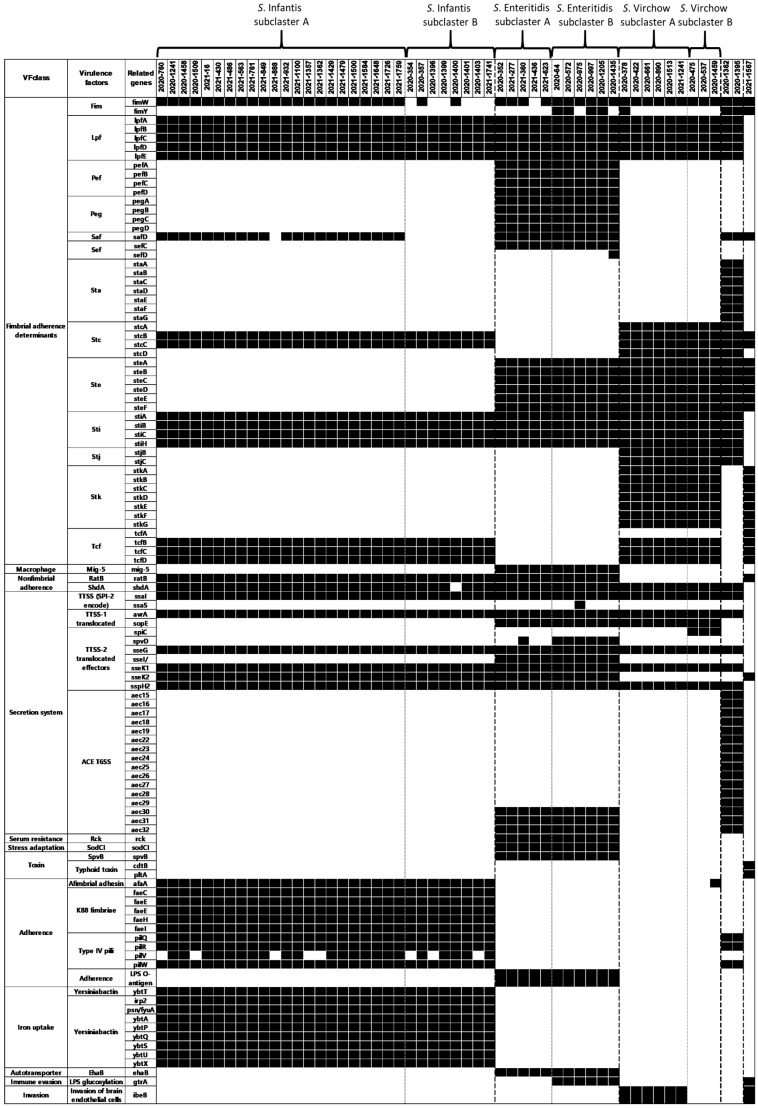
Identified virulence factor genes of multidrug-resistant *Salmonella* spp. except common virulence factor genes.

In total, 230 pertinent genes belonging to 14 virulence factor classes were detected, namely fimbrial adherence determinants, macrophage-inducible genes, magnesium uptake, non-fimbrial adherence determinants, regulation, secretion system, serum resistance, stress adaptation, toxin, adherence, iron uptake, autotransporter, immune evasion, and invasion. The majority of genes belonged to fimbrial adherence determinants (94/230) and secretion systems (98/230). In total, 120 genes were identified (Table 3), of which 110 had different virulence gene profiles (Figure 4). Several genes were dominant in each serotype. For example, in the fimbrial adherence determinant virulence factor class, eight (*pefABCD* and *pegABCD*) and seven (*staABCDEFG*) genes were predominant in *S*. Enteritidis and *S*. Agona, respectively. Iron uptake genes (*irp2*, *psn/fyuA*, and *ybtAPQSTUX*) were exclusively found in *S*. Infantis. There were clear differences in several VFs, even between subclusters of the same serotype, such as the secretion system (*spvD*) and immune evasion (*gtrA*) in *S*. Enteritidis; the secretion system (*spiC/ssaB*) and invasion (*ibeB*) in *S*. Virchow; and fimbrial adherence determinants (*fimW* and *safD*) in *S*. Infantis.

## 4. Discussion

Recently, *S*. Infantis carrying the pESI-like megaplasmid has been reported worldwide [20,21,22]. Several studies have reported that the presence of pESI or pESI-like megaplasmids increases antibiotic resistance and toxin levels in *S*. Infantis [23]. *Salmonella* Infantis carrying the pESI plasmid was reportedly predominant in feces and dust from commercial broiler farms in Korea [24]. *Salmonella* Infantis was the most frequently identified serovar in eggs, and pESI-like megaplasmids have been identified in the broiler industry [25,26]. The pESI plasmid has the potential to spread *S*. Infantis carrying the pESI plasmid to the community in a short period of time [20,21,27]. Despite these reports, the genetic analysis and timing of the spread of *S*. Infantis remain unclear. Therefore, we performed a WGS analysis of MDR Salmonella isolated from Korean poultry meat in 2020–2021, earlier than the previous report. The analysis results showed that *S*. Infantis carrying the pESI plasmid was isolated. In addition to *S*. Infantis, *S*. Enteritidis and *S*. Virchow were shown as the major MDR *Salmonella* in Korean poultry.

In this study, 53 MDR *Salmonella* spp. isolates were identified from 1232 poultry samples (723 chicken and 509 duck meat samples) collected in five areas of South Korea from 2020 to 2021. Of the 53 strains, 94% (50/53) were serotypes (*S*. Infantis, *S*. Enteritidis, and *S*. Virchow) typically found in poultry in South Korea [28,29,30]. *Salmonella* Infantis, *S*. Enteritidis, and *S*. Virchow, belonging to ST32, ST11, and ST16, respectively, showed the same results as those reported previously [31,32]. Most *Salmonella* showed clearly different homology depending on the source of isolation, even though they were the same serotype and sequence type. However, in *S*. Infantis subcluster A strains, 21 strains were sourced from chicken meat and one from duck meat, suggesting that cgST267045 may have been transmitted from chickens to ducks or as a result of contamination in meat processing.

In this study, various antibiotic resistance, plasmid, *Salmonella* pathogenicity island, mobile genetic element, and virulence factor genes were detected and clustered into similar types according to serotype and cgST. *S*. Infantis was divided into two subclusters based on cgST; however, the genetic difference was not clear. The IncFIB (pN55391) plasmid replicon was detected in all *S*. Infantis. Extended-spectrum beta-lactamase (ESBL)-producing *S*. Infantis carrying an IncFIB(pN55391)-like plasmid was first isolated in Israel and quickly disseminated worldwide [27,33,34]. Recently, *S*. Infantis carrying the pESI-like megaplasmid was reported for the first time in eggs in Korea in 2022 [24]. IncFIB(pN55391) was one of the replicons typical of the “parasitic” pESI-like megaplasmid found [24]. In this study, *S*. Infantis carrying the IncFIB(pN55391) showed resistance to various antimicrobial subclasses (tetracycline, beta-lactam, aminoglycoside, sulfonamide, phenicol, trimethoprim, and disinfectant), consistent with other reports [9,24,25,33]. This study used samples from 2020 to 2021, earlier than the previously reported 2022 [24,25,26]. *S*. Infantis carrying the IncFIB(pN55391) has not been reported among *Salmonella* isolates from poultry samples prior to 2020 in South Korea [10,11,35]. To the best of our knowledge, this study is the earliest time to isolate *S*. Infantis carrying the IncFIB (pN55391) in Korea. Therefore, we suspect the time when the new *S*. Infantis carrying the IncFIB(pN55391) began spreading was in 2020. 

Contrastively, *S*. Enteritidis and *S*. Virchow subclusters showed different genetic profiles. The ABR gene profile of the *S*. Enteritidis subcluster A was common among MDR *S*. Enteritidis isolated from the chicken industry in South Korea [11,35]. *Salmonella* with a genetic profile similar to that of *S*. Enteritidis subcluster B was also isolated from Chinese ducks and is a strong candidate to be the major MDR *Salmonella* in ducks [36]. Both subclusters carried the beta-lactam resistance gene (*bla*_CTX-M-15_ and *bla*_TEM-1B_), but in the 3rd and 4th clusters, cephalosporin (ceftiofur, ceftazidime, and cefepime) resistance was clearly different. Additionally, a marked disparity in gentamicin resistance was observed, which was *S*. Enteritidis subclass A carrying the *aac(3)-IId* gene [37]. Both subclusters carried IncFIB(S) and IncFII(S), but only subcluster B contained the IncX1 plasmid. IncX1 is reported to carry beta-lactam, aminoglycoside, and sulfonamide resistance genes [38,39], which is consistent with our study. In addition, Tn2 in all cluster B strains carried *bla*_TEM-1B_, and one strain was detected with the composite transposon cn_35009_IS26 that also carried *aph(6)-ld*, *aph(3*″*)-lb*, *sul2*, and *bla*_TEM-1B_ with the IncX1 plasmid. Therefore, the composite transposon cn_35009_IS26 was a strong candidate for increasing the antibiotic resistance threat of duck-isolated Salmonella by carrying four AMR (*bla*_TEM-1B_, *aph(3*″*)-Ib*, *aph(6)-Id*, and *sul2*) and IncX1 plasmid genes. No mobile colistin resistance genes were detected in *S*. Enteritidis despite the fact that most *S*. Enteritidis have colistin resistance. Colistin-resistant mcr-negative *S*. Enteritidis may be associated with chromosomal mutations, such as those in components of lipopolysaccharide and outer membrane synthesis and modification (*RfbN*, *LolB*, *ZraR*) and the multidrug efflux pump (*MdsC*) [40]. The *S*. Virchow cluster showed extremely different ABR genetic profiles between subclusters and had the lowest ARG presence. The *S*. Virchow cluster A was resistant to ampicillin, ceftiofur, cefoxitin, nalidixic acid, streptomycin, and tetracycline, consistent with several reports [29,35]. Subcluster B carried only two ABR genes, with *bla*_CMY-2_ being unique among the 53 strains, conferring resistance to amoxicillin/clavulanic acid, including penicillin and cephalosporin. Although *bla*_CMY-2_ is a plasmid-mediated gene [41,42], no promising plasmid candidates were detected in the current study.

*Salmonella* chromosomes and plasmid regions encoding virulence-related genes, such as those involved in invasion, survival, and extraintestinal spread, are named SPIs [43]. In this study, 11 *Salmonella* SPIs (SPI1, SPI2, SPI3, SPI4, SPI5, SPI9, SPI10, SPI13, SPI14, C63PI, and CS54) were identified, and the SPI profiles were found to be identical at the serotype cluster level. SPI1, SPI2, SPI3, SPI4, and SPI5, which are more critical for *Salmonella* pathogenesis than other SPIs [9], were commonly identified among the *Salmonella* strains tested. SPI9 was also one of the most common SPIs in the current study, encoding a type 1 system similar to SPI4 [43]. In this study, the three common MDR serotypes (*S*. Infantis, *S*. Enteritidis, and *S*. Virchow) may have been influenced by SPI-13 and SPI-14, which promoted the colonization of chicken spleen [44]. In total, 230 virulence factor genes were identified, with 120 genes related to five virulence factor classes (fimbrial adherence determinants, macrophage-inducible genes, magnesium uptake, non-fimbrial adherence determinants, regulation, and secretion system) being common in this study. In contrast, 47.8% of the virulence factor-related genes showed different virulence gene profiles among the serotype clusters. In particular, diverse genes from two virulence factor classes (attachment and iron uptake) have been identified in *S*. Infantis. The diverse attachment factor genes may play distinct roles in chick infection [45], and a robust iron uptake system may contribute to *Salmonella* fitness and pathogenicity in vivo, potentially allowing rapid dissemination [46].

This study has several limitations. Due to the nature of the poultry industry in Korea, we were unable to identify which farm the meat samples originated from. If we could identify which farm a particular sample originated from, we would be able to have a more in-depth discussion. The study design was limited to bacterial selection for WGS analysis. Of the 408 *Salmonella* isolates (198 from chicken and 210 from duck meat), 53 strains with high levels of MDR were selected and analyzed using WGS. *Salmonella* isolates sensitive to antimicrobials were excluded from the study. Therefore, the distribution of *Salmonella* serotypes in South Korean poultry may differ from those observed in this study. To address these limitations, increasing the number of WGS analyses to cover all *Salmonella* isolates would provide greater insight into the traceback investigation of *Salmonella* from Korean poultry. 

Our study suggests that *S*. Infantis carrying the pESI plasmid first emerged in Korea in 2020 and quickly became the major serotype in the poultry industry in 2022. This new major serotype in the Korean poultry industry spreads rapidly and carries a large number of lethal virulence genes. Therefore, we analyzed the genetic characteristics of MDR *Salmonella* from the Korean poultry industry, including *S*. Infantis carrying the pESI plasmid. Ultimately, this study may help to increase the understanding of MDR *Salmonella* in poultry meat in Korea and to help control its spread.

## Figures and Tables

**Table 1:** Sample tested in this study.

Meat Type	Region					
	Seoul and Gyeonggi-do	Gyeongsang-do	Chungcheong-do	Jeolla-do	Gangwon-do	Total
Chicken	197	319	83	104	20	723
Duck	151	195	92	62	9	509
Total	348	514	175	166	29	1232

**Table 2:** Antimicrobials tested in this study.

Antimicrobial Subclasses	Antimicrobial Agents	Range Tested
Aminoglycosides	Gentamicin	1–64
	Streptomycin	16–128
Aminopenicillin	Ampicillin	2–64
β-lactam/β-lactamase inhibitor combinations	Amoxicillin/Clavulanic acid	2/1–32/16
Cephamycin	Cefoxitin	1–32
Cephalosporin III	Ceftiofur	0.5–8
	Ceftazidime	1–16
Cephalosporin IV	Cefepime	0.25–16
Carbapenem	Meropenem	0.25–4
Fluoroquinolone	Ciprofloxacin	0.12–16
Folate pathway inhibitors	Trimethoprim/Sulfamethoxazole	0.12/2.38–4/76
	Sulfisoxazole	16–256
Phenicols	Chloramphenicol	2–64
Polymyxins	Colistin	2–16
Quinolone	Nalidixic acid	2–128
Tetracyclines	Tetracycline	2–128

**Table 3:** Identified common virulence factor genes of multidrug-resistant *Salmonella* spp.

VF Class	Virulence Factor	Related Gene
Fimbrial adherence determinants	Agf/Csg	*csgA*, *csgB*, *csgC*, *csgD*, *csgE*, *csgF*, *csgG*
	Bcf	*bcfA*, *bcfB*, *bcfC*, *bcfD*, *bcfE*, *bcfF*, *bcfG*
	Fim	*fimA*, *fimC*, *fimD*, *fimF*, *fimH*, *fimI*, *fimZ*
	Saf	*safB*, *safC*
	Stb	*stbA*, *stbB*, *stbC*, *stbD*, *stbE*
	Std	*stdA*, *stdB*, *stdC*
	Stf	*stfA*, *stfC*, *stfD*, *stfE*, *stfF*, *stfG*
	Sth	*sthA*, *sthB*, *sthC*, *sthD*, *sthE*
Macrophage inducible genes	Mig14	*mig14*
Magnesium uptake	Mg^2+^ transport	*mgtB*, *mgtC*
Non-fimbrial adherence determinants	MisL	*misL*
	SinH	*sinH*
Regulation	PhoPQ	*phoP*, *phoQ*
Secretion system	TTSS (SPI1 encode)	*hilA*, *hilC*, *hilD*, *iacP*, *iagB*, *invA*, *invB*, *invC*, *invE*, *invF*, *invG*, *invH*, *invI*, *invJ*, *orgA*, *orgB*, *orgC*, *prgH*, *prgI*, *prgJ*, *prgK*, *sicA*, *sicP*, *sipD*, *spaO*, *spaP*, *spaQ*, *spaR*, *spaS*, *sprB*
	TTSS (SPI2 encode)	*ssaC*, *ssaD*, *ssaE*, *ssaG*, *ssaH*, *ssaJ*, *ssaK*, *ssaL*, *ssaM*, *ssaN*, *ssaO*, *ssaP*, *ssaQ*, *ssaR*, *ssaT*, *ssaU*, *ssaV*, *sscA*, *sscB*, *sseB*, *sseC*, *sseD*, *sseE*, *ssrA*, *ssrB*
	TTSS effectors translocated via both systems	*slrP*
	TTSS1 translocated effectors	*sipA*, *sipB*, *sipC*, *sopA*, *sopB/sigD*, *sopD*, *sopE2*, *sptP*
	TTSS2 translocated effectors	*pipB2*, *pipB*, *sifA*, *sifB*, *sseF*, *sseJ*, *sseL*

## Data Availability

All WGS data on the 53 isolates are available under NCBI BioProject PRJNA1105733.
